# Posterolateral corner of the knee: a systematic literature review of current concepts of arthroscopic reconstruction

**DOI:** 10.1007/s00402-020-03607-z

**Published:** 2020-09-21

**Authors:** Sebastian Weiss, Matthias Krause, Karl-Heinz Frosch

**Affiliations:** Department of Trauma and Orthopaedic Surgery, University Medical Center Hamburg-Eppendorf, Martinistr. 52, 20246 Hamburg, Germany

**Keywords:** Knee, Posterolateral corner, Popliteus, Lateral collateral ligament, Reconstruction, Arthroscopy

## Abstract

**Introduction:**

Injuries of the posterolateral corner (PLC) of the knee lead to chronic lateral and external rotational instability and are often associated with PCL injuries. Numerous surgical techniques for repair and reconstruction of the PLC are established. Recently, several arthroscopic techniques have been published in order to address different degrees of PLC injuries through reconstruction of one or more functional structures. The purpose of this systematic review is to give an overview about arthroscopic techniques of posterolateral corner reconstructions and to evaluate their safeness.

**Materials and methods:**

A systematic review of the literature on arthroscopic reconstructions of the posterolateral corner of the knee according to the PRISMA guidelines was performed using PubMed MEDLINE and Web of Science Databases on June 15th, 2020. Inclusion criteria were descriptions of surgical techniques to reconstruct different aspects of the posterolateral corner either strictly arthroscopically or minimally-invasive with an arthroscopic assistance.

**Results:**

Arthroscopic techniques differ with regard to the extent of reconstructed units (popliteus tendon, popliteofibular ligament, lateral collateral ligament), surgical approach (transseptal, lateral) and biomechanical results (anatomic vs. non-anatomic reconstruction, restoration of rotational instability and/or lateral instability).

**Conclusion:**

Different approaches to arthroscopic PLC reconstruction are presented, yet clinical results are scarce. Up to now good and excellent clinical results are reported. No major complications are reported in the literature so far.

## Introduction

### Anatomy of the posterolateral corner

The posterolateral corner of the knee has a complex anatomic composition, which was only thoroughly discovered in recent years and has since gained increased focus in diagnostics and treatment. It consists of the lateral collateral ligament (LCL) and the popliteus complex (PTC). The popliteus complex itself contains the popliteus muscle tendon unit (PLT) and the arcuate complex (AC), which is formed by the popliteofibular ligament (PFL), the fabellofibular ligament and the popliteomeniscal fibers [1].

In its complexity, the posterolateral corner is essential to stabilization against various forces to the knee. The arcuate complex with its most prominent part, the popliteofibular ligament, serves primarily as a static stabilizer against external tibial rotation [2]. Together with the popliteus muscle tendon unit, which also functions as a dynamic stabilizer against external rotation, the arcuate complex prevents posterior tibial translation [1, 3, 4]. In PCL injuries, if a side-to-side difference in posterior drawer larger than 12 mm is detected, an additional injury to the PLC is highly probable, indicating its important role as a dorsal stabilizer [5]. Respectively, the lateral collateral ligament is the most important stabilizer against varus forces [3].

### Injuries to the posterolateral corner

Subsequently, if one or more components of the posterolateral corner are injured, their loss of function leads to different degrees of instability. A widely used classification was described by Fanelli and Larson in 2002. While Type A injuries only show a rotational instability, types B and C are defined by an additional lateral instability against varus forces (B = slight varus relaxation of 5–10°; C = severe varus relaxation > 10°) [6–8].

Based on biomechanical examinations, the instability of the posterolateral corner can be differentiated in dorsal instability, which is mainly caused by the PCL, lateral (varus) instability due to injury to the lateral collateral ligament (LCL) and rotational instability, which is mainly linked to lesions of the popliteus complex [3]. This results in a modified classification of dorsolateral instabilities (Fig. 2):

Type 1: isolated posterior instability through isolated injury of the PCL.

Type 2: posterolateral rotational instability without lateral instability (PCL and the popliteus complex are injured, the LCL is intact, no arthroscopic gutter drive through sign).

Type 3: posterolateral rotational instability with varus instability (PCL, the popliteus complex and the LCL are injured, positive arthroscopic gutter drive through sign).

Type 4: posterolateral rotational instability with gross varus instability (PCL, the popliteus complex, the LCL and additional structures (such as the iliotibial band, biceps tendon, posterolateral capsule, etc.) are injured).

Injuries to the PLC are critically underdiagnosed, yet they are reported to be present in almost 16% of knee injuries [9]. Especially in cases of posterior cruciate ligament injuries they are often overlooked, despite up to 70% of PCL injuries showing concomitant damage to the PLC [1, 10]. Untreated PLC injuries can lead to chronic pain, instability with shifted biomechanics of the knee and therefore early development of osteoarthritis [9] and may also cause failure of surgically isolated reconstructed cruciate ligaments [7].

### Treatment of PLC injuries

To address these injuries, a broad spectrum of surgical techniques has been described, ranging from repairs to reconstructive techniques and anatomic versus non-anatomic procedures [11–14]. In Fanelli Type C chronic injuries (> 3 weeks), anatomic reconstruction has been described and established as the most optimal treatment [15, 16]. In this type of injury additional refixation of the iliotibial band and/or biceps tendon is necessary and therefore usually needs open surgery.

In recent years, novel arthroscopic reconstruction techniques have been developed, addressing different aspects of the PLC, especially to treat type 2 and 3 injuries (Fig. 2). Advantages of arthroscopic surgery over open surgery include better visualization of anatomical landmarks, which are hidden in open procedures, lower infection rates, lesser amounts of scar tissue, less pain, faster rehabilitation and especially a better protection of the peroneal nerve since its visualization and preparation is obsolete [1].

In this comprehensive review, these recently emerged arthroscopic reconstruction techniques of the posterolateral corner are described, summarized and compared.

## Materials and methods

This study followed the guidelines of the Preferred Reporting Items for Systematic Review and Meta-Analysis (PRISMA) statement [17]. A systematic review of the literature on arthroscopic reconstructions of the posterolateral corner of the knee was performed using PubMed MEDLINE and Web of Science Databases on June 15th, 2020. The following search terms were used: “posterolateral reconstruction” OR “arthroscopic posterolateral reconstruction” OR “plc reconstruction” OR “posterolateral corner reconstruction” OR “posterolateral corner” AND “arthroscopy” OR “popliteus reconstruction” OR “arthroscopic popliteus reconstruction” OR “popliteus” AND “arthroscopy”.

Inclusion criteria were descriptions of surgical techniques to reconstruct different aspects of the posterolateral corner either strictly arthroscopically or minimally-invasive with an arthroscopic assistance.

Exclusion criteria were descriptions of open surgical procedures, clinical studies without detailed description of the surgical procedure, case reports and non-English language articles.

Bibliographies of included articles were screened for potentially missed articles.

**Fig. 1:**
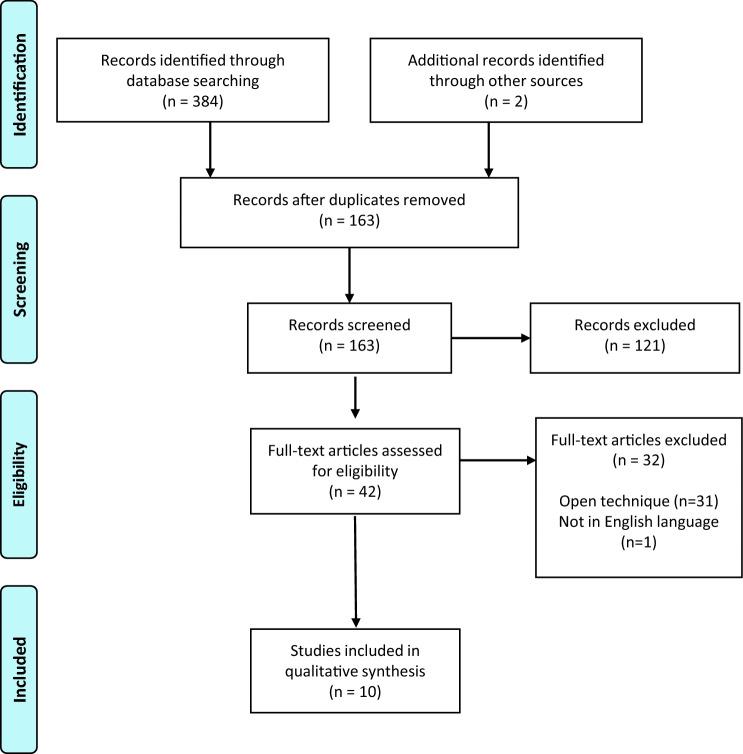
PRISMA flow diagram outlining the study selection process

A total of 386 articles was identified, after removal of duplicates (*n* = 223), 163 titles and abstracts were screened for eligibility by two independent reviewers. After exclusion of 121 articles through screening, all 42 remaining articles underwent a full-text search by the reviewers to evaluate matching of inclusion and exclusion criteria. Any discrepancies were mutually resolved. Ultimately, 10 articles were included (Fig. 1).

Since this review compares surgical techniques and no clinical or biomechanical results, data for statistical analysis was not available.

## Arthroscopic techniques for plc reconstruction

### Sling reconstruction of the popliteus tendon (PLT)

Feng et al. published an all-arthroscopic technique for a non-anatomical reconstruction of the popliteus tendon using a semitendinosus tendon graft [18].

They first depicted the femoral PLT footprint through an anterolateral and superior anterolateral/parapatellar portal, with a subsequent placement of a drill tunnel in the center of the femoral PLT footprint. Next, through transseptal visualization of the posterolateral corner via a posteromedial portal, the posterior horn of the lateral meniscus was detached from the posterior capsule, displaying the popliteus muscle and its musculotendinous junction. Introducing an ACL tibial drill guide through the posterolateral portal and targeting the popliteal musculotendinous junction area, a tibial drill tunnel was created. The tendon graft was fixed to the femoral drill tunnel first, then to the tibial tunnel, closely following the native popliteus tendon.

The authors described their early clinical results as promising, including 6 cases in which restoring external tibial rotation stability was as effectively managed as with comparable open techniques. Further follow-up data or detailed results are not available.

### Popliteus bypass graft

**Fig. 2:**
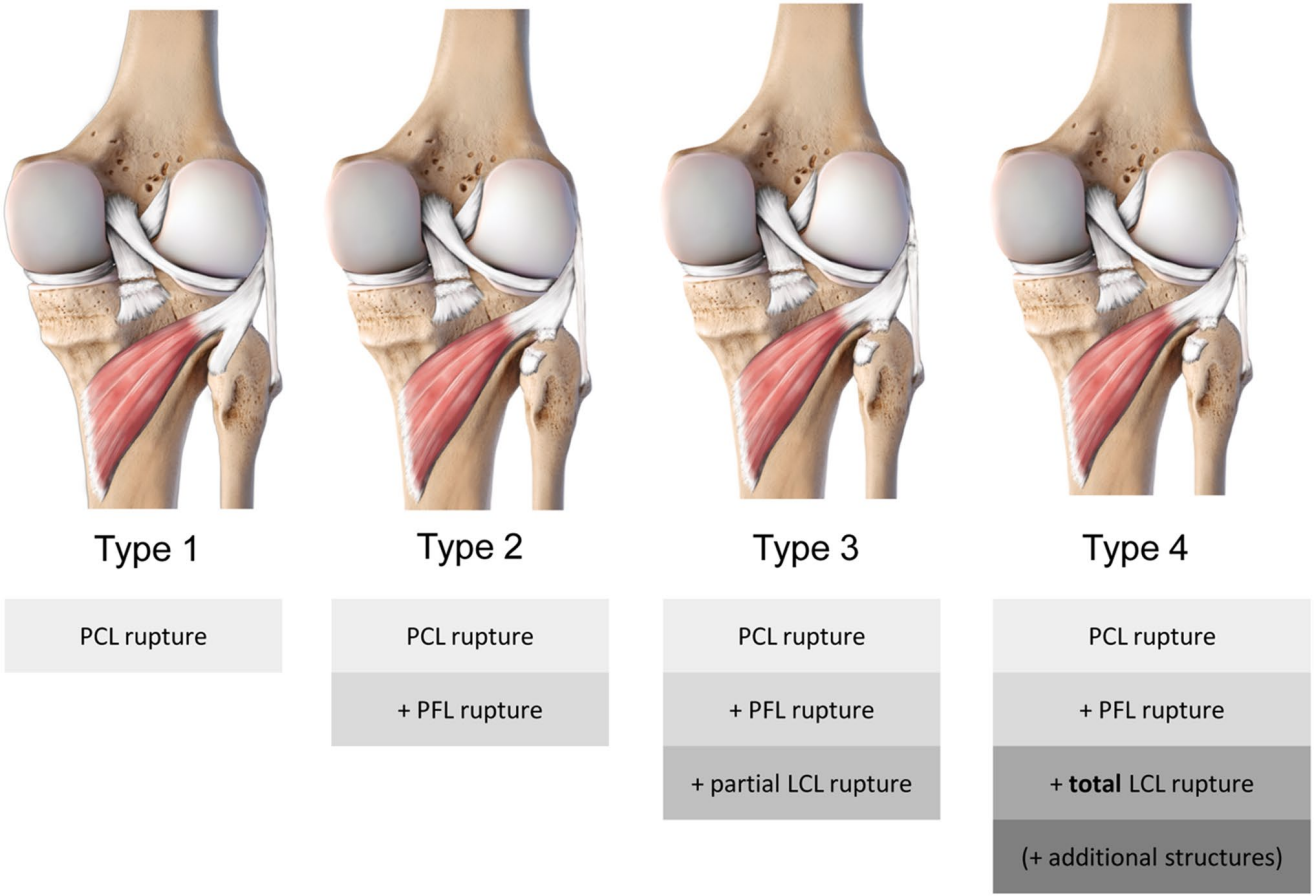
Schematic drawings of the posterolateral corner of the knee and classification of instabilities with regard to injured structures involved. Type 1: Dorsal instability caused by PCL rupture. Type 2: Dorsal and rotatory instability through PCL rupture and PFL rupture. Type 3: Dorsal, rotatory and (minor) lateral instability through additional partial LCL rupture. Type 4: Dorsal, rotatory and (major) lateral instability through total LCL rupture and additional structures (i.e. iliotibial band, biceps tendon, posterolateral capsule, etc.)

An anatomic arthroscopic reconstruction of the static function of the popliteus complex called “popliteus bypass graft” was described by Frosch et al. [1]. In a cadaver study, they developed a method for arthroscopic anatomical reconstruction of the static stabilizing function of the popliteus tendon, in order to provide a suitable treatment for Fanelli Type A (Type 2, Fig. 2) instabilities.

Six arthroscopic portals were used, including a posteromedial and posterolateral access as a transseptal approach to visualize the popliteus tendon and its tibial sulcus [19]. By introducing a drill guide from the anteromedial portal through the knee, the tibial drill tunnel was aimed at the distal third of the sulcus popliteus under direct visualization.

For the femoral drill tunnel, a high anterolateral and parapatellar lateral portal were used to visualize the femoral footprint of the PLT, which was subsequently placed in its center. A single- or double-stranded hamstring tendon graft with a length of 11–12 cm was pulled through and fixed to the tibia and femoral lateral condyle.

In a second step, drill tunnel placement was evaluated with regard to 9 anatomical landmarks after removing the soft tissue around the knee. Both the tibial and femoral drill tunnel placements showed high accuracy with less than 3 mm deviation from their defined anatomical locations [20].

In 2016, Frosch et al. presented clinical results of the first 19 patients to undergo surgery for a popliteus bypass graft in combination with PCL reconstruction for Type 2 injuries. There were no technique-related complications, side-to-side difference in posterior drawer was reduced from 13.3 [± 1.9] mm (preoperative) to 2.9 [± 2.2] mm and dial test was negative in 10 out of 12 patients at 1-year follow-up with a mean Lysholm Score of 88.4 (± 8.7) points [21].

### Fibula-based reconstruction technique to control rotational instability

Another approach to address rotational instabilities through anatomic reconstruction of the popliteofibular ligament was presented by Song et al. [22]. In contrast to Frosch et al., their technique is based on a fibular drill tunnel.

Through a standard anterolateral portal, an accessory lateral gutter portal is established close to the femoral PLT footprint. The femoral tunnel is drilled in the footprint’s center with a 6–7 mm drill to a depth of 25 mm.

To establish the fibular drill tunnel, the authors also rely on the transseptal approach described by Ahn et al. [19]. After identification of the popliteus musculotendinous junction, the posterior horn of the lateral meniscus is detached from the posterior capsule to gain visualization of the proximal tibiofibular joint and the fibular PFL insertion site. By using an ACL tibial guide, a tunnel (6 mm) is drilled from the anterior fibular cortex towards the posterior fibular head at the site of the PFL insertion. A semitendinosus graft (alternatively tibialis anterior allograft) is then passed through the fibular tunnel from anterior to posterior and towards the femoral socket, where it is fixed with a bio-absorbable screw (7 mm). Finally, after tensioning, the graft is fixated with an interference screw (7 mm) aiming towards the posterior fibular aperture at 30° of knee flexion and neutral rotation.

The authors describe the use of this technique in a single patient, who had suffered an anteromedial strike to the knee with subsequent dorsal (addressed by PCL reconstruction) and rotational instability. Two years after surgery his posterior drawer (3.8 mm vs. 11.8 mm preoperative) and external rotation (4° vs. 16° preoperative) showed satisfying results and he had resumed all sporting activities.

### Stabilization of the posterolateral joint capsule

In 2017, Ohnishi et al. described an all-arthroscopic technique for non-anatomic reconstruction in cases of isolated posterolateral rotational instability [23]. They described a stabilization of the posterolateral joint capsule through tightening and attachment of the lateral meniscus to the lateral tibial plateau. This procedure was deemed suitable for patients with chronic posterolateral rotatory instability (PLRI) in the absence of significant injuries to the PCL, popliteus tendon/PFL or LCL.

Through a midlateral portal (exact location is unspecified), the rim of the lateral tibia plateau is abraded and two suture anchors are placed ventral and dorsal of the popliteal hiatus. Subsequently, sutures are passed through the popliteomeniscal fibers and posterolateral joint capsule from inferior to superior and the capsule is tightened.

The authors report promising short-term results in rectifying isolated PLRI, with the disadvantage of a non-anatomical reconstruction and limitation of the lateral meniscus’ normal motion. Specifics of their short-term results or long-term results were not published.

### Fibula-based anatomic PLC reconstruction (technique according to Arciero)

Frings et al. devised the first all-arthroscopic PLC reconstruction for a combined rotational and lateral instability (Type 3, Fig. 2) [24]. This technique was inspired by an open surgical procedure described by Arciero [25], which has shown good biomechanical and clinical results [26, 27].

In this technique, similar to the popliteus tendon bypass graft by Frosch et al. [1], a transseptal display of the posterolateral corner through a posteromedial portal is key, since it enables exposure of the posteromedial fibular surface under direct visualization. After preparation of the fibular head, a drill tunnel is established with the help of an aiming device in an anterolateral to posteromedial direction under arthroscopic vision.

Thereafter, femoral PLT and LCL footprints were visualized through a high anterolateral and parapatellar portal and drill tunnels for femoral graft fixation were placed in the footprint’s center. Then, an armed gracilis or semitendinosus tendon graft with a minimum length of 20 cm was fixed to the femoral PLT footprint first and subsequently shuttled along the PLT’s native course, through the fibular drill tunnel from posteromedial to anterolateral and finally attached to the femoral LCL footprint drill tunnel. Fixation of the graft is performed by a bio interference screw in the fibula and in the femur in different flexion angles as well.

Ahn et al. described a modification of this technique in 2019 [28]. Additionally to a transseptal approach, an accessory inferior posterolateral portal is established just above the posterosuperior aspect of the fibular head to create a stab incision into the PFL attachment site on the popliteus tendon. The fibular drill tunnel is placed similarly to Frings et al. Femoral drill tunnels are positioned at the same entry points, but are placed convergently to create a communicating bony tunnel. The tendon graft is passed through this subcortical femoral tunnel which divides it into a PLT and an LCL portion. The LCL graft portion is passed through the fibular drill tunnel from anterolateral to posteromedial. Next, the PLT graft portion is pulled along the PLT native course, through a loop wire attached to the musculotendinous junction of the PLT and ultimately through the fibular drill tunnel from posteromedial to anterolateral. Lastly, the graft ends are fixated to the fibular tunnel with two interference screws from both tunnel ends at 30° of knee flexion.

Liu et al. described another similar technique in 2020 [29], but instead of using a transseptal approach, arthroscopic visualization of the posterolateral corner is enabled by establishing a posterolateral portal 5 mm above and posterior to the tip of the fibular styloid under arthroscopic visualization from a high lateral portal at the femoral PLT footprint, so this visualization is likely restricted in comparison to a transseptal view Through the posterolateral portal the fibular PFL insertion site is exposed after radiofrequency debridement. Tunnel drilling is executed in a similar manner with an ACL aiming device, then the tendon graft is secured to the fibular head first with a 6 × 23 mm bioabsorbable screw and subsequently fixed to the femoral LCL drill tunnel at 0° of knee flexion and the femoral PLT tunnel at 30° of flexion.

The authors conducted a biomechanical evaluation of their arthroscopic reconstruction, which showed significant reduction of varus, external rotational and posterior instability at 15° and 30° of flexion, resulting in no significant differences in stability between reconstructed and intact knees. Clinical results of this technique are still missing.

### Arthroscopic popliteus tenodesis

A technique to arthroscopically repair posterolateral injuries by performing a popliteus tenodesis with the advantage of using a native, vascularized material was designed by Hermanowicz et al. [30]. Due to its nature of re-establishing the static stabilization against external rotation, this procedure is restricted to treating Fanelli Type A injuries as well as patients with an intact femoral PLT attachment.

For ideal visualization, the authors created a midlateral portal with the knee in full extension, located 1.5 cm above the fibular head and 1 cm anterior to the lateral collateral ligament. The site for tenodesis is consistent with the dorsolateral tibial attachment site described by Frosch et al. in the sulcus popliteus [1], yet the distal/ventral portion of the tibial tunnel is located medially below the pes anserinus.

After attaching a multi-strand polyethylene suture to a ventral button, tension of the PLT tenodesis is regulated until arthroscopic signs of PLC injuries (drive-through sign, elevation of lateral meniscus) are eliminated.

Despite some limitations (no extension deficit allowed, risk of popliteus tendon or lateral meniscus injury, additional lateral collateral ligament reconstruction required in Type 3 (Fig. 2) injuries), the authors describe their procedure as very efficient and reproducible without exhausting additional treatment options. Up to now no biomechanical or clinical data exist in a reliable number of patients. No complications with this technique were described.

### Arthroscopic-assisted anatomic PLC reconstruction

To address patients with higher grade instabilities (Type 3, Fig. 2), Hermanowicz et al. described an arthroscopic-assisted fibula-/tibia-based combined reconstruction of PLC and LCL in 2019 [31]. Reconstructing both ligaments required two autologous tendon grafts (semitendinosus tendon for PLT, gracilis tendon for LCL).

To do so, the authors established a high midlateral portal in addition to the priorly described midlateral portal. First, a tibial drill tunnel is positioned in the known location of the sulcus popliteus to the ventromedial tibial cortex. Through the high midlateral portal, the femoral footprint of the PLT is exposed and a transfemoral tunnel is drilled to the medial epicondyle. To complete PLC reconstruction, the semitendinosus tendon graft is shuttled through the femur, then along the popliteus tendon’s native course and finally through the tibial drill tunnel, directed from the popliteus musculotendinous junction area towards the ventromedial tibial cortex with subsequent tensioning and fixation to the tibia.

Reconstruction of the LCL is facilitated by two larger incisions (4–5 cm horizontal above femoral LCL attachment, 2–3 cm vertical above fibular head). After sectioning of the iliotibial band and drilling of a femoral tunnel at the site of LCL attachment, focus is set on the fibular attachment. Here, the authors promote a drill tunnel reaching from the middle of and perpendicular through the fibular head towards the medial aspect of the tibia, just below the MCL distal attachment. Hence, the channel goes directly through the tibiofibular joint, which might be a disadvantage of this technique. By channeling the gracilis graft from the medial tibial cortex to the medial femoral cortex, it does not only reconstruct function of the LCL, but also tibiofibular stability.

### Tibia and Fibula-based anatomic PLC reconstruction (technique according to LaPrade)

Another method for reconstruction of higher grade posterolateral instabilities (Fanelli Type B/C) through an all-arthroscopic fibula-/tibia-based technique was described by Kolb et al. [32]. Their arthroscopic procedure follows the well-established open reconstruction described by LaPrade et al. [12].

To accomplish visualization of the posteromedial surface of the fibular head and the popliteal sulcus, a transseptal portal is created consistent with the approach by Frosch et al. [21] and Frings et al. [24]*.* By placing a cannulated aiming device through the posterolateral portal on the medial fibular surface under direct vision, a fibular drill tunnel is directed from anterolateral (LCL attachment site) to posteromedial (PFL attachment site) and subsequently reamed to a 5–6 mm tunnel. After introducing a marking hook through the knee from the anteromedial portal [21], a tibial drill tunnel (7 mm) is directed from the anterolateral cortex (center between tibial tuberosity and Gerdy’s tubercle) to the distal third of the popliteal sulcus of the posterolateral tibia.

By using an arthroscopic shaver through a lateral parapatellar portal, femoral LCL and PLT footprints are exposed. Two guide pins are drilled in parallel orientation towards proximal medial and reamed to a 5–6 mm diameter tunnel with a minimum distance of 5 mm between tunnels. Since reconstruction requires the use of two tendon grafts, the authors recommend a semitendinosus tendon with a minimum length of 28 cm.

The popliteus bypass graft (min. 11 cm) is attached to the femur, then shuttled along the popliteus tendon native path and through the tibia. After femoral attachment, the second graft (min. 17 cm) is passed under the iliotibial band to the anterolateral fibular tunnel, through the fibula and ultimately through the tibial tunnel from posterior to anterior. The LCL graft is fixed at 20° of knee flexion in the fibular tunnel and the popliteus bypass graft at 70° of knee flexion in the tibial tunnel, both with biointerference screws.

In this technique, rotational and lateral instabilities are addressed, similar to Hermanowicz et al. [31], with the striking advantage of sparing the tibiofibular joint.

## Comparison of techniques

The common advantages of all arthroscopic PLC reconstructions in comparison to open procedures could be reduced infection rates, less postoperative pain, faster rehabilitation, less scar tissue formation and a more aesthetical result. Furthermore, arthroscopic surgeries have the striking advantage of sparing the common peroneal nerve of preparation and visualization, hence reducing its risk of injury. Past anatomic studies have shown, that establishment of a posterolateral portal is safe in a knee flexion angle of 90°, as this position creates the largest distance to the common peroneal nerve (25.4 mm ± 9.2 mm according to Ahn et al. [33] and 26.6 mm ± 9.5 mm according to Makridis et al. [34]). Additionally, arthroscopic display of the knee joint ensures visualization of key structures which would usually remain hidden in open procedures or could only be visible after extensive soft tissue preparation.

Up to now there is only one study which showed that arthroscopic reconstructions of the PLT achieve similar subjective and objective outcomes as open reconstructions [35]. Open procedures for reconstruction in higher grade instabilities especially in techniques according to Arciero [26, 36] and LaPrade [37] have shown significantly improved objective and subjective stability in clinical studies [38] and are well-established. The techniques described by Frings et al. [24] and Liu et al. [29] present the arthroscopic equivalents for open Arciero reconstructions and the technique described by Kolb et al. [39] displays an arthroscopic version of LaPrade’s procedure. Clinical results of these techniques remain elusive and will have to be evaluated and compared to open procedures.

All of the above listed surgical techniques differ from each other in various aspects (Table 1). As for the ultimately most important result, the clinical outcome, only very little information is published. Unfortunately, most studies have not (yet) followed up their proposed surgical techniques with differentiated short- or long-term results in patients who were treated accordingly. Frosch et al. are one of few working groups who have presented specific results, in clinical outcome [21] as well as through radiological evaluation of drill tunnel placements [20].

**Table 1:** Overview of arthroscopic techniques for PLC reconstruction

	Feng 2009	Frosch 2014	Song 2015	Ohnishi 2017	Frings 2018	Hermanowicz 2018	Hermanowicz 2019	Kolb 2019	Ahn 2019	Liu 2020
Anatomic		X	X		X	X	X	X	X	X
Non-anatomic	X			X						
Fibula-based			X		X		X	X	X	X
Tibia-based	X	X		X		X	X	X		
PLT restored	X	X	(X)		X	X	X	X	X	X
LCL restored					X		X	X	X	X
Repair						X				
Reconstruction	X	X	X	X	X		X	X	X	X
Transseptal approach	X	X	X		X			X	X	

True characteristics for each technique marked with “X”

Since clinical results of other techniques remain elusive, the different approaches can only be compared with regard to technical issues. Most techniques present an anatomical reconstruction [1, 22, 24, 28–32] and most procedures are tibia-based, while the reconstructions depicted by Song et al., Frings et al., Ahn et al. and Liu et al. are solely fibula-based techniques [22, 24, 28, 29]. When looking at the addressed structures, the only methods with a combined arthroscopic targeting of PLT and LCL are by Frings et al., Hermanowicz et al., Kolb et al., Ahn et al. and Liu et al. [24, 28, 29, 31, 32], other procedures only target the PLT, which leaves concomitant LCL injuries to open reconstructions (Table 2) [10, 11, 20].

**Table 2:** Degrees of instability (Type 1–4, Fig. 2), addressed by each technique

	Type 1	Type 2	Type 3 and 4
Feng 2009	Addressed through additional PCL reconstruction	✔	✖
Frosch 2014		✔	✖
Song 2015		✔	✖
Ohnishi 2017		✖	✖
Frings 2018		✔	✔
Hermanowicz 2018		✔	✖
Hermanowicz 2019		✔	✔
Kolb 2019		✔	✔
Ahn 2019		✔	✔
Liu 2020		✔	✔

✔ = suitable; ✖ = non-suitable

There is a dividing line regarding portal use and development, leading to differences in arthroscopically exposed or hidden and therefore endangered structures. One critical aspect of most arthroscopic techniques is the transseptal approach, proposed by Feng et al., Frosch et al., Song et al., Frings et al., Kolb et al. and Ahn et al. [1, 18, 22, 24, 28, 32]. This approach, enabled through the creation of a posteromedial portal, demands working in the posterior compartment of the knee, with an increased risk of injury to the neurovascular bundle [40]. Therefore, only experienced surgeons in the field of knee arthroscopy should perform these procedures. Assuming meticulous protection of the neurovascular bundle, a transseptal approach allows for significantly optimized visualization of the posterolateral corner, which ultimately leads to a more precise procedure. Another critical point of fibula based arthroscopic techniques is the arthroscopic exposure of the posteromedial facet of the fibula head. In this area the peroneal nerve is around 2 cm distally to the fibular drill tunnel in a 90° flexed knee.

Ohnishi et al., Hermanowicz et al. and Liu et al. developed their techniques without the use of a transseptal approach, claiming that therefore an advanced surgical skill is not required [31]. Their midlateral portal on the other hand puts the native PLT, the LCL and the lateral meniscus at risk of being injured. In the author’s opinion, despite the sparing of a transseptal approach, these procedures should nonetheless only be performed by arthroscopically experienced knee surgeons. No neuro-vascular complications have been described with the different arthroscopic techniques for posterolateral corner reconstruction up to now.

## Conclusion

With the increasing attention drawn towards treatment of posterolateral corner injuries, various arthroscopic techniques have emerged, which have not yet been subject to extensive evaluation for their clinical outcome. While authors tend to describe their techniques as promising, patient results have yet to be obtained. Taking all of the before-mentioned differences into account, clinical studies will have to show which procedures provide the best treatment for patients with injuries to the posterolateral corner. Nevertheless, arthroscopic techniques seem to be promising and may have the potential to develop as a standard procedure in posterolateral corner reconstruction. Up to now no neurovascular injuries have been described.
